# Impact of WPA’s Telepsychiatry Global Guidelines on Clinical Practice, International Collaboration, and Education

**DOI:** 10.1192/j.eurpsy.2022.442

**Published:** 2022-09-01

**Authors:** D. Mucic

**Affiliations:** Little Prince Treatment Centre, Telepsychiatry, Copenhagen V, Denmark

**Keywords:** telepsychiatry, collaboration, education, skills and competencies

## Abstract

**Introduction:**

Telepsychiatry is the best-documented e-Mental Health application. It refers to the use of videoconferencing in the provision of mental health services. During the COVID19 pandemic, in response to physical distancing, mental health services worldwide have turned to online consultations. For the vast majority of clinicians, it was the first time they use telepsychiatry, and very few have received training in how to do it.

**Objectives:**

- to present the main objectives and messages of the WPA Global Guidelines for Telepsychiatry related to competencies & skills, educational & legislative needs, and international collaboration.

**Methods:**

A structured review of the main challenges, innovations, and settings in the first Global Telepsychiatry Guidelines, published by WPA in February 2021.

**Results:**

The benefits of increased access to telehealth services are apparent for telepsychiatry, but benefits can only be realized if the tools are used by clinicians who have the appropriate training and guidance. With proper preparation and thoughtful risk management, telepsychiatry can be an invaluable tool for allowing greater access to care. However, certain prerequisites must be fulfilled to achieve the desired goals. These prerequisites are e.g. choice of the technology, settings, patient/provider preferences as well as competencies and skills described in this document.

**Conclusions:**

The need for training among health care professionals is the highest priority. The urgent need for clinical training and skills building around e-mental health inclusive telepsychiatry, will determine the influence that psychiatry can have in addressing the mental health sequelae of the COVID19 pandemic via competent practice and increased international collaboration.

**Disclosure:**

I am the main author of “WPA Telepsychiatry Global Guidelines”

